# Visual performance with multifocal lenses in young adults and presbyopes

**DOI:** 10.1371/journal.pone.0263659

**Published:** 2022-03-17

**Authors:** Shrilekha Vedhakrishnan, Maria Vinas, Clara Benedi-Garcia, Pilar Casado, Susana Marcos

**Affiliations:** 1 Laboratory of Visual Optics & Biophotonics, Instituto de Optica, (IO-CSIC), Consejo Superior de Investigaciones Cientificas, Madrid, Spain; 2 Wellman Center for Photomedicine, Massachusetts General Hospital, Harvard Medical School, Boston, Massachusetts, United States of America; 3 Center for Visual Sciences, The Institute of Optics, Flaum Eye Institute, University of Rochester, Rochester, New York, United States of America; Nicolaus Copernicus University, POLAND

## Abstract

A better understanding of visual performance with Multifocal Contact Lenses (MCLs) is essential, both in young eyes, where MCLs may be prescribed to control the progression of myopia wherein the MCLs optics interact with accommodation, and in presbyopes, where MCLs are increasingly used to compensate the lack of accommodation. In this study, we evaluated the through focus visual acuity (TFVA) with center-near MCLs of three additions (low, medium and high) and without an addition (NoLens) in 10 young adults and 5 presbyopes. We studied the effect of accommodation, age and pupil diameter (in cyclopleged subjects) on visual performance. The MCLs produced a small but consistent degradation at far (by 0.925 logMAR, averaged across eyes and conditions) and a consistent benefit at near in young subjects with paralyzed accommodation (by 1.025 logMAR), and in presbyopes with both paralyzed and natural accommodation (by 1.071 logMAR, on average). TFVA in young adults with NoLens and all MCLs showed statistically significant differences (Wilcoxan, p<0.01) between natural and paralyzed accommodation, but not in presbyopes with MCLs. In young adults, VA improved with increasing pupil diameter with the HighAdd MCL (0.08 logMAR shift from 3 to 5-mm pupil size). Visual imbalance (standard deviation of VA across distances) was reduced with MCLs, and decreased significantly with increasing near add. The lowest imbalance occurred in young adults under natural accommodation and was further reduced by 13.33% with MCLs with respect to the NoLens condition. Overall, the visual performance with MCLs in young adults exceeds that in presbyopes at all distances, and was better than 0.00 logMAR over the dioptric range tested. In conclusion, the center-near lenses do not degrade the near high contrast visual acuity significantly but maintains the far vision in young adults, and produce some visual benefit at near in presbyopes.

## Introduction

Myopia is one of the most common eye disorders, with increasing prevalence worldwide and considered as a significant global health problem [1]. A variety of interventions have been proposed to halt myopia progression. Among them, optical treatments have been favored to date to slow down myopia, based on the rationale that retinal blur is the visual signal that drives refractive error development. Among those optical approaches, Multifocal Contact Lenses (MCLs), which work under the principle of simultaneous vision, i.e., projecting an image focused simultaneously at far and near, have been increasingly used in the last years [2].

Presbyopia, the age-related loss of the crystalline lens’s ability to accommodate dynamically from far to near, affects 100% of the population over the age of 45. Simultaneous Vision in the form of MCLs (as well as Multifocal Intraocular Lenses (MIOLs)) are increasingly used for correcting presbyopia. These multifocal corrections aim at restoring the near vision functionality lost in the presbyopic eye, while still correcting vision at far. Different studies [3–6] in presbyopic patients corrected with MCLs or MIOLs report measurements of through-focus (TF) visual performance revealing a gain at near, generally accompanied with visual degradation at far. Understanding the factors contributing to the performance of MCLs in presbyopes, including lens design, pupil diameter and residual accommodation is important to improve patient management and select the appropriate correction.

In progressive myopia, two hypotheses have been postulated for justifying an intervention with MCLs. The first relies on the associations found between myopia and increased accommodative lag [2, 7–9]. The presence of a near addition (regardless of where it is located on the lens), which produces a focus at near, may reduce hyperopic defocus resulting from the accommodative lag, thereby potentially reducing a strong signal for eye growth. Typically, lenses used for myopia progression control have a center-distance design. However, clinically, benefits of MCLs with center-near design have also been shown in individuals with a large lag of accommodation and near esophoria [10]. The second hypothesis for the principle of operation of MCLs to treat myopia relies on observations that the peripheral retina plays a role in proper emmetropization [11–13]. In particular, the peripheral hyperopic defocus present in myopic eyes as a result of myopic eye globe shape and by correction with conventional spherical lenses is thought to produce a stimulus for eye growth [11]. Based on this hypothesis, MCLs would slow down myopia progression by producing myopic peripheral defocus that counteracts the natural hyperopic defocus and thereby the stimulus for eye growth. However, the role of the peripheral retina in myopia progression has been contested in several studies, that either failed to find an impact of peripheral defocus on foveal axial elongation or statistical differences in the retinal shape of emmetropes or myopes [14, 15].

The center-distance multifocal lenses are expected to create peripheral myopic defocus for far distances, although it has been observed that the effect is reduced at near [16]. In fact, the effects of the center-distance lenses on the periphery are not as large as expected, as they produce myopic defocus primarily in one meridian but not the entire retinal periphery [16]. Surprisingly, a study has found myopic peripheral defocus even in eyes with center-near multifocal lenses [17], likely reflecting large inter-subject variability in the shape of the peripheral retina. The mechanism of the MCLs may therefore not entirely depend on modulating the sign of defocus in the periphery as this can be variable. While peer-reviewed literature on myopia progression control with MCLs primarily reports the use of center-distance lens designs [17, 18], there is also anecdotal evidence that center-near lens designs have been effective at slowing down myopia progression [10, 17].

The extent to which MCLs interact with accommodation has been explored in some studies. Petterson et al. [19] found similar accommodation in pre-presbyopic adults when subjects were wearing bifocal center-distance corrections as compared to their lag with single vision lenses, suggesting that young subjects do not relax their accommodation with multifocal aspheric contact lenses. However, this is in contrast with the results of a study on accommodation dynamics with induced spherical aberration (using adaptive optics), which showed that accommodative lag is modulated differently by the presence of positive spherical aberration (equivalent to center- distance add) or negative spherical aberration (equivalent to center-near add) [20]. That study showed that the presence of negative spherical aberration reduced accommodative lag while positive spherical aberration in fact increased accommodative lag, which may favor the use of center-near addition lenses. Interestingly, Theagarayan et al. [21] used contact lenses that induced either positive or negative spherical aberration and found the same result, i.e. adding negative spherical aberration (unlike positive spherical aberration) improves the slope of the accommodation stimulus-response curve and decreases lag of accommodation, again suggesting that center-near MCLs would favor a more accurate accommodative response. Regardless of the mechanism of operation, the presence of a near addition, either central or peripheral, seems to produce a sufficiently strong inhibitory signal to slow down myopia progression, hence exploring visual performance in young adults fitted with center-near addition lenses is interesting, as there are a larger variety of center-near lenses commercially available.

In presbyopes, center-near lenses are more popular than center-distance, as comparative studies have shown a higher performance of center-near lenses [22, 23]. One could expect that the optical design of MCLs, except for minor adjustments, may be rather similar for both target populations (young adults and presbyopes). A major fundamental difference between the two groups is that in young adults, when prescribed with MCLs, accommodation is functional, at least to a much larger extent than in presbyopes. Also, pupil diameter in a young population is generally larger than in older eyes [24, 25]. However, in both groups, a potential detriment to the use of MCLs is the unwanted visual compromise resulting from simultaneous vision, which has been evaluated primarily in studies on presbyopic subjects [26]. To date, understandably, most studies in young adults wearing MCLs evaluate their success in controlling myopia progression [27–30], and only a few studies have thoroughly evaluated visual performance and visual quality with MCLs [3, 31, 32]. In fact, differences in visual performance with MCLs are expected to depend on several lens design factors (i.e., the power profile of the lenses, near add [33, 34] and the patient’s optical profile, including pupil size [34, 35] and optical aberrations). To our knowledge, no previous study has undertaken a systematic evaluation of the influence of lens design, natural aberrations, pupil diameter and presence/absence of accommodation on TF visual performance with MCLs.

In this study, we evaluated TF visual performance with commercially available center-near multifocal designs of three different magnitudes of addition (low/medium/high) at different foci, both with natural and paralyzed accommodation. The study was performed in a group of young adults and a group of presbyopes.

## Methods

Through focus visual acuity (TFVA) was measured in two groups of subjects with different refractive profiles (young adults and presbyopes), under natural conditions, both with natural and paralyzed accommodation and for different pupil diameters. The measurements were made with the MCLs on the eye and without the MCLs (NoLens) as a control.

### Subjects

15 subjects of European origin participated in the study, clustered in two groups: (1) 10 young adults, average age: 26.9±2.2, average spherical equivalent -2.2±0.8D (2) 5 presbyopes, average age: 53±2.0; average spherical equivalent 0.25±0.9D. Table 1 shows the individual patients’ profile. All subjects were familiar with the insertion and removal of contact lenses, being either habitual or occasional soft contact lens wearers.

**Table 1:** Individual refractive profile of the subjects of the two groups (young adults and presbyopes).

Subject	Group	Age (yrs)	Gender	Measured Eye	Spherical error (D)	Astigmatism (D)	Astigmatism axis (deg)	Duration: Habitual (H)/Occasional (O) lens wearer
**S1**	**YOUNG ADULTS**	24	F	OD	-1.25	-0.5	70	O (SCL)
**S2**		24	F	OD	-2.5	-	-	H (SCL)
**S3**		24	M	OD	-4.5	-	-	O (SCL)
**S4**		24	F	OD	-4	-	-	O (SCL)
**S5**		25	F	OD	-0.5	-	-	O (SCL)
**S6**		26	M	OD	0	-	-	O (SCL)
**S7**		26	F	OD	-4.5	-	-	H (SCL)
**S8**		27	F	OD	-1.5	-	-	H (SCL)
**S9**		31	F	OD	-1.75	-0.5	90	O (SCL)
**S10**		38	M	OD	0	-0.5	108	O (SCL)
**S11**	**PRESBYOPES**	47	F	OD	1.5	-	-	O (MCL)
**S12**		52	F	OD	2.5	-	-	H (MCL)
**S13**		53	F	OD	-2.75	-	-	H (MCL)
**S14**		55	M	OD	0	-	-	O (MCL)
**S15**		58	M	OD	0	-	-	H (MCL)

ID, age, gender, measured eye (dominant eye in all cases), spherical error, astigmatism, astigmatism axis, and duration of lens wear: habitual (H) or occasional (O) of the MCL (Multifocal lens) and SCL (for standard soft CLs).

The study protocols met the tenets of the Declaration of Helsinki, and had been approved by the CSIC Institutional Review Boards. All participants were informed about the study and experimental procedures and signed informed consent prior to the experimental session.

### Multifocal Contact lenses (MCLs)

The MCLs used in this study was 1-Day Acuvue Moist MCLs (Johnson and Johnson Vision Care, Jacksonville, FL, USA) [36, 37]. The lens is a soft lens, daily disposable, and has a center-near aspheric profile. All subjects were fitted with a -2D for far, with three different additions: Low (+1.25D), Medium (+1.75D) and High (+2.50D). A previous study with the same MCLs showed that the depth of focus increased with the increasing addition of the lenses [36]. The subject’s residual refractive error was compensated with a motorized Badal system.

### Experimental set-up

Measurements were performed in a custom-developed Adaptive Optics (AO) system, described in detail previously [38]. The system has a Hartmann-Shack wavefront sensor (32x32 microlenses; HASO 32 OEM, Imagine eyes, France) and an electromagnetic deformable mirror (52 actuators and 50mm stroke; MIRAO, Imagine eyes, France), which, for the purpose of this study, were only used to measure and correct the system’s aberrations in a closed-loop operation. A motorized Badal system corrects and induces defocus, while a pupil monitoring system (LED ring illuminator and a CCD camera) is used to align and monitor the position of the subjects. Visual stimulus was displayed in a CRT monitor (Mitsubishi Diamond Pro2070) with an angular subtend of 2 deg in the psychophysical channel. All optoelectronic elements are controlled in the computer by C++ and MATLAB software. A 7-mm artificial pupil was placed in a conjugate pupil plane in the visual stimulus channel, which allowed measurements with different pupil diameters (5, 4 and 3 mm).

### Experimental procedure

Each subject was measured in two different sessions. The first session was performed under cycloplegia with 1% Tropicamide; (Alcon Cusi, Barcelona, Spain) (2 drops prior to the experiment, and repeated every hour). The second session was performed on non-cyclopleged eyes. Both sessions involved TFVA measurements for 4 conditions (LowAdd, MediumAdd, HighAdd real MCLs in the eye, and without an MCL in the eye (NoLens)). In session 1, TFVA measurements were performed for 3 different pupil diameters (5, 4 and 3 mm), with the pupil size being controlled by the artificial pupil of the system. In session 2, measurements were done only for the natural pupil (i.e., not limited by the artificial pupil in the system). Each session was performed on different days (separated by 1–2 days or less). The duration of session 1 was 7–8 hours, with frequent breaks. The duration of session 2 was approximately 4 hours. The subjects received the contact lenses at the beginning of the session, inserted them and were allowed to settle for 10–15 min prior to the start of the measurements. The subjects were not informed about the addition of the MCLs.

All measurements were performed monocularly (dominant eye) in a darkened room. Before starting the measurements, subjects were instructed on the nature of the experiment and performed some trial runs. The subject was then asked to adjust the Badal system position to achieve the best focus for far, for each condition (NoLens, LowAdd, MediumAdd, HighAdd). The zero-defocus setting for each condition was the average of five best-focus readings. Deviations in the best focus with MCLs from the NoLens did not vary more than 0.375/0.50/0.50D across conditions (LowAdd/MediumAdd/HighAdd), consistent with the expected focus shift produced by these MCLs [36, 39] and confirming that the far focus (and not a near focus) was always selected for distance vision.

### Through focus visual acuity (TFVA)

Visual acuity (VA) measurements were performed for each condition at best focus, and in 0.5 D steps around best focus, covering a full dioptric range of 4 D (-3.00D to +1.00D). VA was measured using an 8-Alternative Forced Choice (8AFC) [40] procedure with Tumbling E letter (Black E-letters on a white background) and a QUEST (Quick Estimation by Sequential Testing) algorithm programmed with the Psychtoolbox package [41] to calculate the sequence of the presented stimulus (letter size and orientation) in the test following the subject’s response. Subjects had to determine the orientation of the E-letter, and the size of the stimulus in the subsequent presentation changed depending on the subject’s response. The QUEST routine for each VA measurement consisted of 35 trails, where the threshold criterion was set to 75%. The threshold VA measurement was estimated as the average of the 10 last stimulus values. VA was expressed in terms of logMAR acuity (log MAR = -log_10_ [decimal acuity]) [42].

### Data analysis

**Fig 1:**
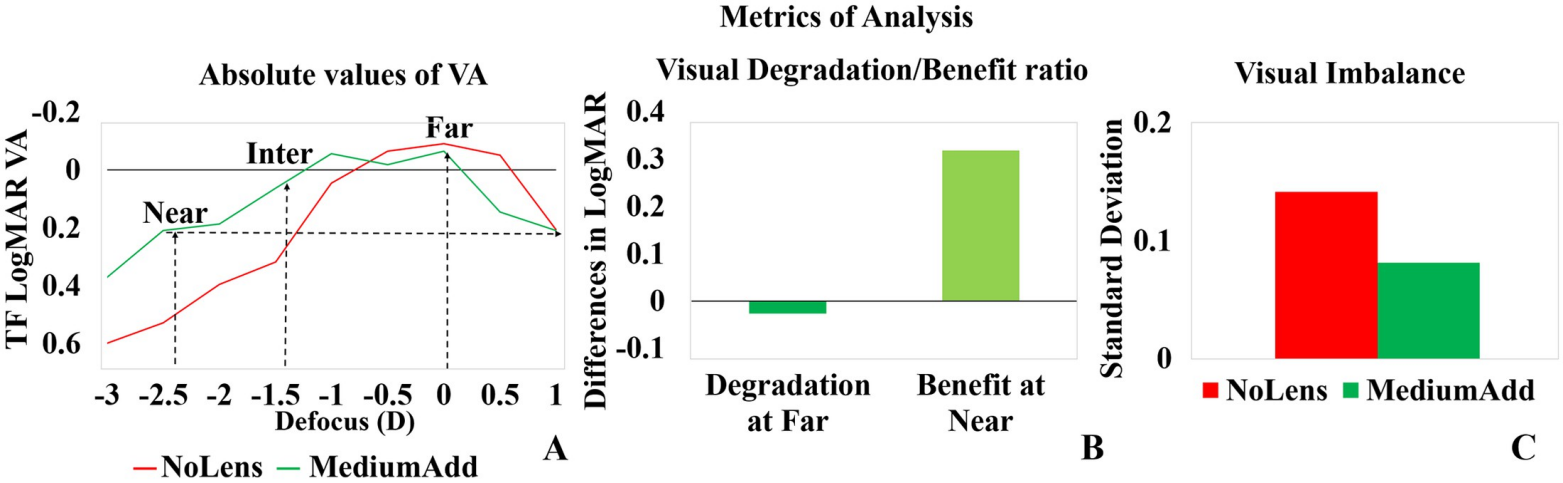
Illustration of different metrics for young subject S#9 with NoLens and MediumAdd CL. (A) TF logMAR VA, highlighting the absolute value of logMAR VA at far (best focus), intermediate (1.5D) and near (2.5D) distances, indicated by vertical dashed lines, and Depth of Focus (defined as the dioptric range where VA is better than 0.2logMAR) indicated by a horizontal line. (B) Differences in logMAR VA for NoLens and MCL at far (Visual Degradation at far, dark green) and at near (2.5D, Visual benefit at near). (C) Visual Imbalance across distances defined as the standard deviation of the logMAR VA values across the TF curve, for the NoLens (Red bar) and MediumAdd CL (Green Bar).

Different metrics of analysis (illustrated in Fig 1) were obtained from the TF curves for each subject and condition, evaluating absolute values of visual performance at different distances (Fig 1A), relative differences in performance with MCL, in comparison with a standard spherical correction (Fig 1B), and the constancy of vision across distances (Fig 1C).

*Absolute VA values* for far (0D), intermediate (1.5D) and near (2.5D) extracted from the TFVA curve as shown in Fig 1(A). These values reflect the visual functionality with the MCLs at different distances.

*Depth-of-focus (DOF)* is defined as the range of defocus over which the VA is 0.2 logMAR or better [43].

*Visual Degradation at far & Visual Benefit at near* were obtained, respectively, as the difference of VA with MCLs relative from VA with NoLens at far (best focus) and at near (+2.50 D), respectively, as shown in Fig 1(B). Specifically, visual degradation at far is defined as (logMAR VA @far (NoLens)–logMAR VA @far (MCLs)). Visual Benefit at Near is defined as (logMAR VA @near (NoLens)–logMAR VA @near (MCLs)). It is expected that MCLs reduce visual performance at far (i.e., negative values) and improve visual performance at near (i.e., positive values). It should be noted that given the logarithmic notation of logMAR differences are translated to ratios in other metrics, such as decimal visual quality or perceptual scorings, and therefore the definitions based on ratios in previous studies are equivalent to those defined here [44].

*Visual Imbalance* is defined as the standard deviation of VA across a TF curve and is illustrated in Fig 1(C). This metric captures the fluctuations in visual performance across distances. For example, VA with a monofocal correction (NoLens) in a patient with paralyzed accommodation, or with a bifocal correction, is expected to vary more across distances (thus resulting in a higher standard deviation, and thus a higher visual imbalance) than with extended depth of focus lenses producing a smoother variation of VA across distances (lower standard deviation, and thus lower imbalance). *Visual constancy* is estimated from visual imbalance following normalization (by a factor of 0.2) and multiplied by -1.

*Overall quality metric*: This metric is the result of the combination of the visual degradation/benefit and visual constancy metric. The overall metric is defined by Visual benefit at near − Visual degradation at far + Visual constancy. A positive value indicates a high overall visual performance while a negative value indicates a degraded visual performance. We evaluated these metrics in all subjects and conditions and studied differences in visual performance across lenses and age groups.

Statistical analysis was performed with SPSS software Statistics 24.0 (IBM, United States). The normality assumption was checked using Shapiro-Wilk’s test. A power analysis (Post hoc analysis) was performed. All the comparisons within the group for each condition had a power value greater than 0.8, indicating a sufficiently large sample. Some comparisons across the two groups (young adults and presbyopes) had a power value of 0.4–0.6 which still indicate significant results. Specific non-parametric tests were used: (1) the Mann-Whitney U test to analyze differences between independent samples (young adults, presbyopes) (2) the Wilcoxon test to analyze differences between two related samples (Accommodation, Visual Benefit and Degradation, Visual Imbalance) within each group, and (3) the Kruskal-Wallis test to analyze differences between NoLens and MCLs conditions. The similarity in the shape of the TF curves between the individual’s native VA and with the MCLs was done using a cross-correlation analysis, with *lag k* and *rho* values representing the largest spike of the series when the elements of both TF curves match exactly and the correlation coefficient, respectively.

## Results

### Visual acuity at far and at near

**Fig 2:**
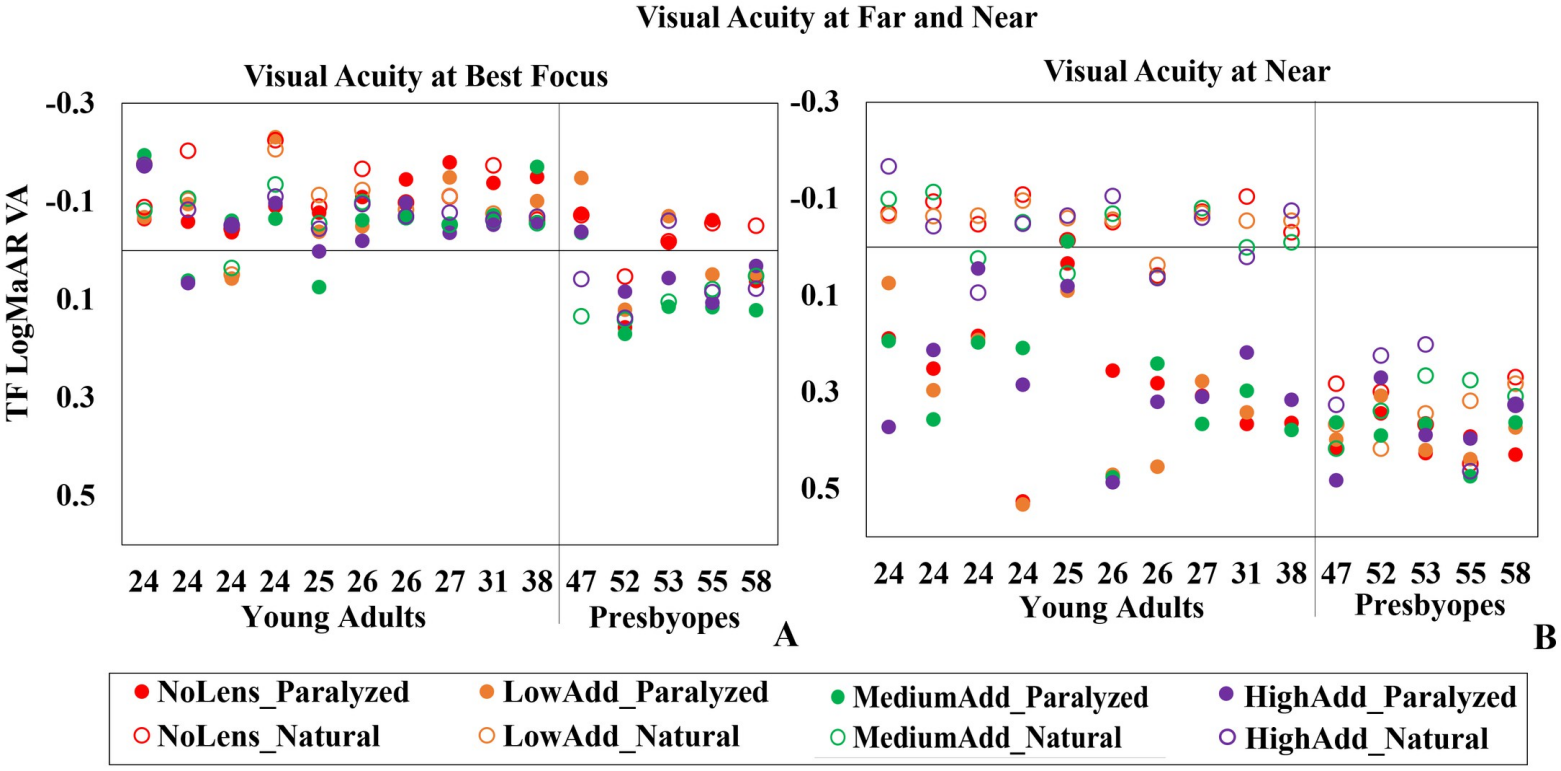
logMAR VA at far and near. (A) logMAR VA at far and (B) logMAR VA at near, for all lenses and conditions, as a function of subject’s age. Different lenses are identified by color—Red: NoLens; Orange: LowAdd; Green: MediumAdd; Purple: HighAdd. Solid symbols stand for paralyzed accommodation and open symbols for natural accommodation. Subjects to the left of the separation bar are young adults; and to the right of the separation bar are presbyopes.

Fig 2(A) shows the VA at far (best focus) and (B) VA at near for all subjects and lenses as a function of age, for both paralyzed (solid symbols) and natural accommodation (open symbols). Different colors represent the different lenses (LowAdd: Orange; MediumAdd: Green; HighAdd: Purple and NoLens: Red). Qualitatively, in the young adults group only three subjects showed logMAR VA at far poorer than 0 with some MCL conditions. Also, in the majority of cases (except subjects 1 and 3, 24 years old) the poorest VA values occurred for Medium and HighAdd MCLs. The best VA at near was obtained with the MCLs in the young adults group. With natural accommodation, the performance of the MCLs (LowAdd/MediumAdd/HighAdd) was statistically significantly different in both young adults (Kruskal-Wallis test, p = 0.011) and presbyopes (Kruskal-Wallis test, p = 0.033) for far.

In presbyopes, statistically significant differences were found in VA between far and near both for the NoLens condition and with all MCLs with both natural and paralyzed accommodation (Wilcoxon signed rank test, NoLens/LowAdd/MediumAdd/HighAdd: p = 0.043 and NoLens/LowAdd/MediumAdd/HighAdd: p = 0.043 for paralyzed and natural accommodation respectively). This indicates that VA at near is compromised in presbyopes, as expected, and that MCLs do not produce an improvement in near vision that matches far vision. In the young adults, VA was statistically significantly different between far and near for the NoLens and with all MCLs (Wilcoxon signed rank test, p = 0.005/0.005/0.007/0.005) with paralyzed accommodation and only for the NoLens condition (Wilcoxon signed rank test, p = 0.007) with natural accommodation.

Under natural accommodation, statistically significant differences in VA were found between young adults and presbyopic groups in all conditions for both far (Mann-Whitney U test, NoLens/LowAdd: p = 0.008; MediumAdd: p<0.001; HighAdd: p = 0.003) and near (Mann-Whitney U test, p<0.001). Under paralyzed accommodation, statistically significant differences between young adults and presbyopes occurred in almost all conditions (Mann-Whitney U test, NoLens (p = 0.013), MediumAdd (p = 0.003), HighAdd (p = 0.019)) for far and only with the NoLens (p = 0.028) for near.

No significant differences were found in presbyopes between VA measured under paralyzed and natural accommodation, as expected due to lack of accommodation and smaller natural pupil diameters. However, statistically significant differences were found in VA measured under paralyzed and natural accommodation in the young adults across all conditions (Wilcoxon signed rank test, NoLens/LowAdd p = 0.005; MediumAdd/HighAdd p = 0.007), as a result of operative accommodation.

### Through focus VA: Effect of lens design and accommodation

#### TFVA—Paralyzed accommodation

**Fig 3:**
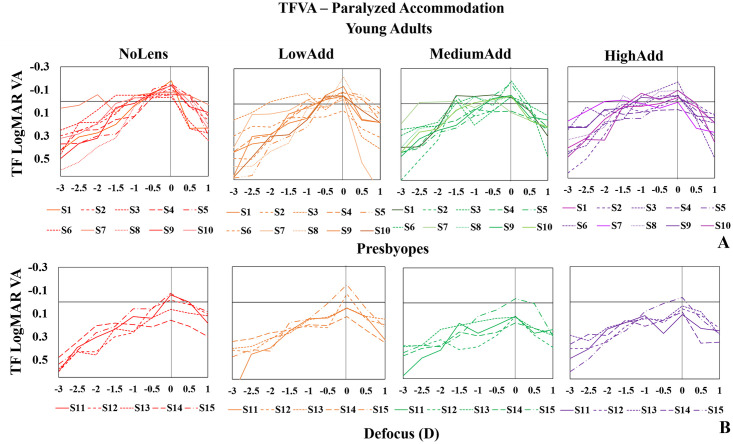
TFVA data shown for all subjects and conditions under paralyzed accommodation with 5 mm pupil diameters. (A) young adults (top panels); (B) presbyopes (bottom panels). Conditions from left to right are NoLens, LowAdd, MediumAdd, HighAdd. Lens condition identified by color—Red: NoLens; Orange: LowAdd; Green: MediumAdd; Purple: HighAdd.

Fig 3 shows TFVA measured under paralyzed accommodation in all young adults (top panels) and all presbyopes (bottom panels) for all 3 lenses (LowAdd: Orange; MediumAdd: Green; HighAdd: Purple) and the NoLens (NoLens: Red) condition, measured with a fixed 5-mm diameter pupil. When comparing the TFVA curves between conditions (NoLens/LowAdd/MediumAdd/HighAdd) within each group, no statistically significant differences were found between groups, indicating that the visual performances were similar across the conditions.

#### TFVA—Natural accommodation

**Fig 4:**
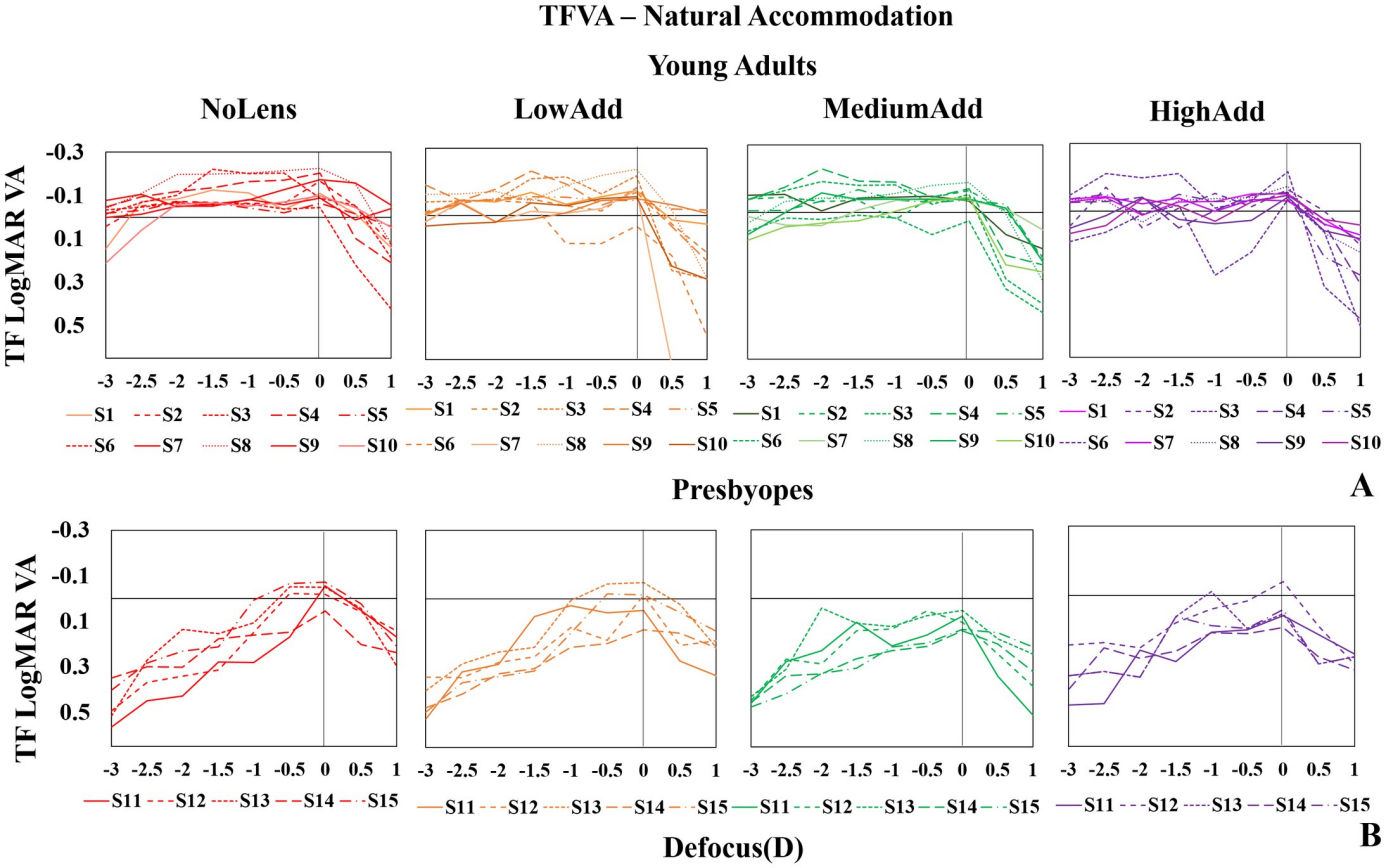
TFVA curves for all subjects and conditions under natural accommodation with natural pupil diameters. (A) young adults (top panels); (B) presbyopes (bottom panels). Conditions from left to right are NoLens, LowAdd, MediumAdd, HighAdd. Lens condition identified by color—Red: NoLens; Orange: LowAdd; Green: MediumAdd; Purple: HighAdd.

Fig 4 shows TFVA measured under natural accommodation (and natural pupil diameter) in all young adults (top panels) and all presbyopes (bottom panels) for all 3 lenses (LowAdd: Orange; MediumAdd: Green; HighAdd: Purple) and the NoLens (NoLens: Red) condition. Unlike under paralyzed accommodation (where both young adults and presbyopes show similar trends, i.e., narrower TFVA with NoLens, which broadens with MCL), TFVA curves are notably different between young adults and presbyopes, indicating a clear effect of accommodation in the young group, and the effect of MCLs in this group is not qualitatively as clear.

Overall, when comparing the paralyzed (Fig 3) and natural accommodation (Fig 4) within each group, no statistically significant differences were found in the presbyopic group. However, in the young adult group, statistically significant differences between paralyzed and natural accommodation were found for all conditions (Wilcoxon signed rank test, NoLens: p = 0.005, LowAdd: p = 0.005, MediumAdd: p = 0.007; HighAdd: p = 0.005) due to the presence of accommodation.

### Intersubject variability

In young adults, intersubject variability in VA at best focus was lowest with NoLens (0.023logMAR) and increased with MCLs with paralyzed accommodation. Overall intersubject variability was higher with paralyzed than with natural accommodation but differences in intersubject variability did not reach statistical significance in either of the groups.

### Average through focus VA

**Fig 5:**
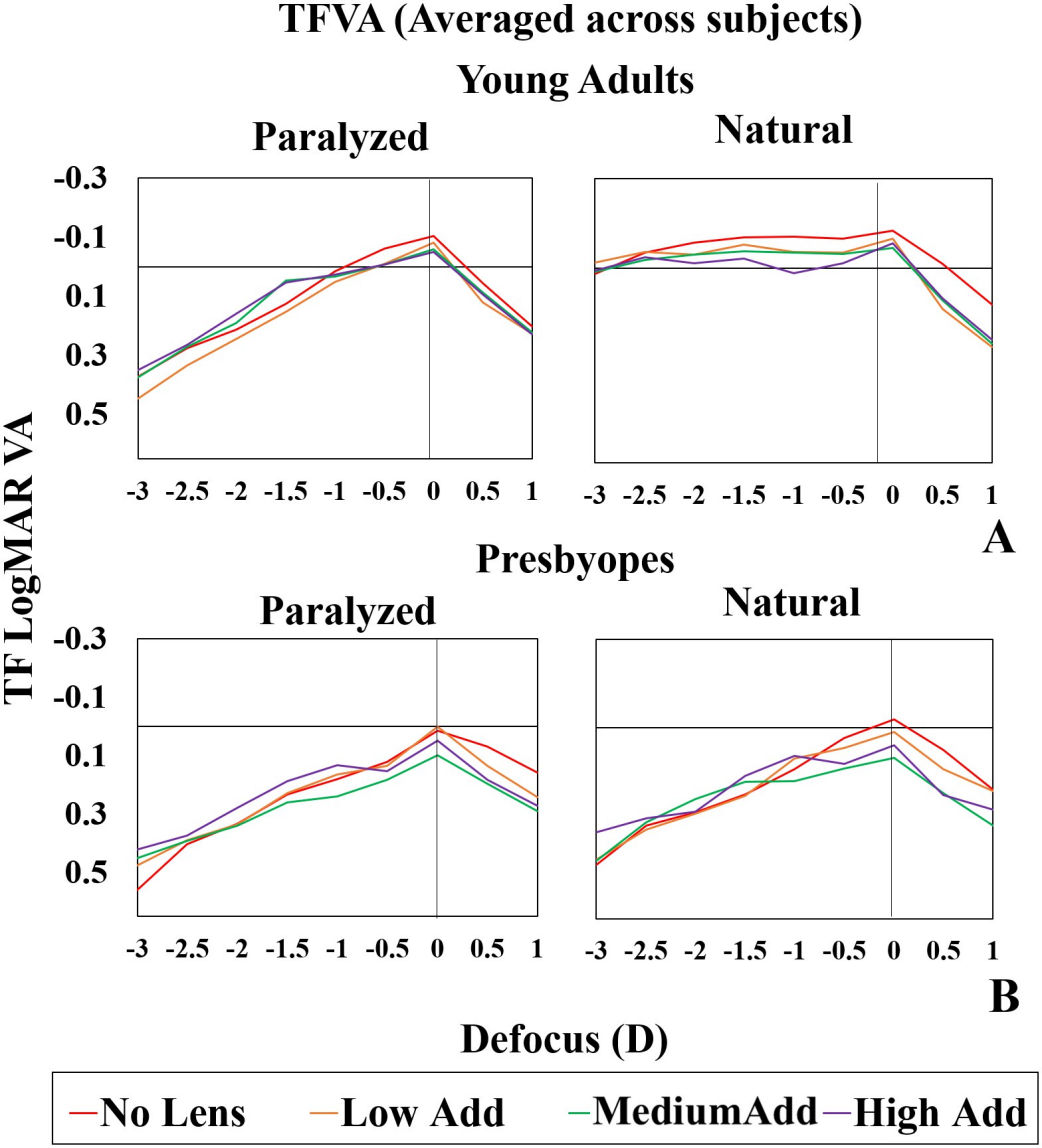
Average TF logMAR VA, across all subjects in the two study groups for paralyzed and natural accommodation. (A) Average TFVA curves for young adults; (B) Average TFVA curves for presbyopes. Left plots: paralyzed accommodation; Right plots: natural accommodation. Each lens condition identified by color—Red: NoLens; Orange: LowAdd; Green: MediumAdd; Purple: HighAdd.

Fig 5 shows TFVA curves averaged across subjects for young adults (A) and presbyopes (B) for paralyzed accommodation (left panels) and natural accommodation (right panels). Each line represents a different lens type (LowAdd: Orange; MediumAdd: Green; HighAdd: Purple and NoLens: Red). In young adults, the shape of the TFVA curves was statistically significantly different between the natural and paralyzed accommodation conditions (cross-correlation: lag k = 0; rho = 0.561 NoLens; rho = 0.204 LowAdd; rho = 0.360 MediumAdd; rho = 0.337 HighAdd). However, in presbyopes, there were no statistically significant differences in the shape of the TFVA curves (cross-correlation: lag k = 0; rho = 0.965 NoLens; rho = 0.979 LowAdd; rho = 0.897 MediumAdd; rho = 0.950 HighAdd), as expected due to largely reduced accommodation.

Medium and HighAdd MCLs improved the performance at intermediate and near vision, compared to the NoLens and LowAdd conditions, under paralyzed accommodation in both groups, and also under natural accommodation in presbyopes. On average, under paralyzed accommodation, DOF increased in young adults from 1.95 D (NoLens) to 2.25 D with the HighAdd, and in presbyopes from 1.4D (NoLens) to 1.5D with the HighAdd lens.

### Through focus VA: Effect of pupil diameter

**Fig 6:**
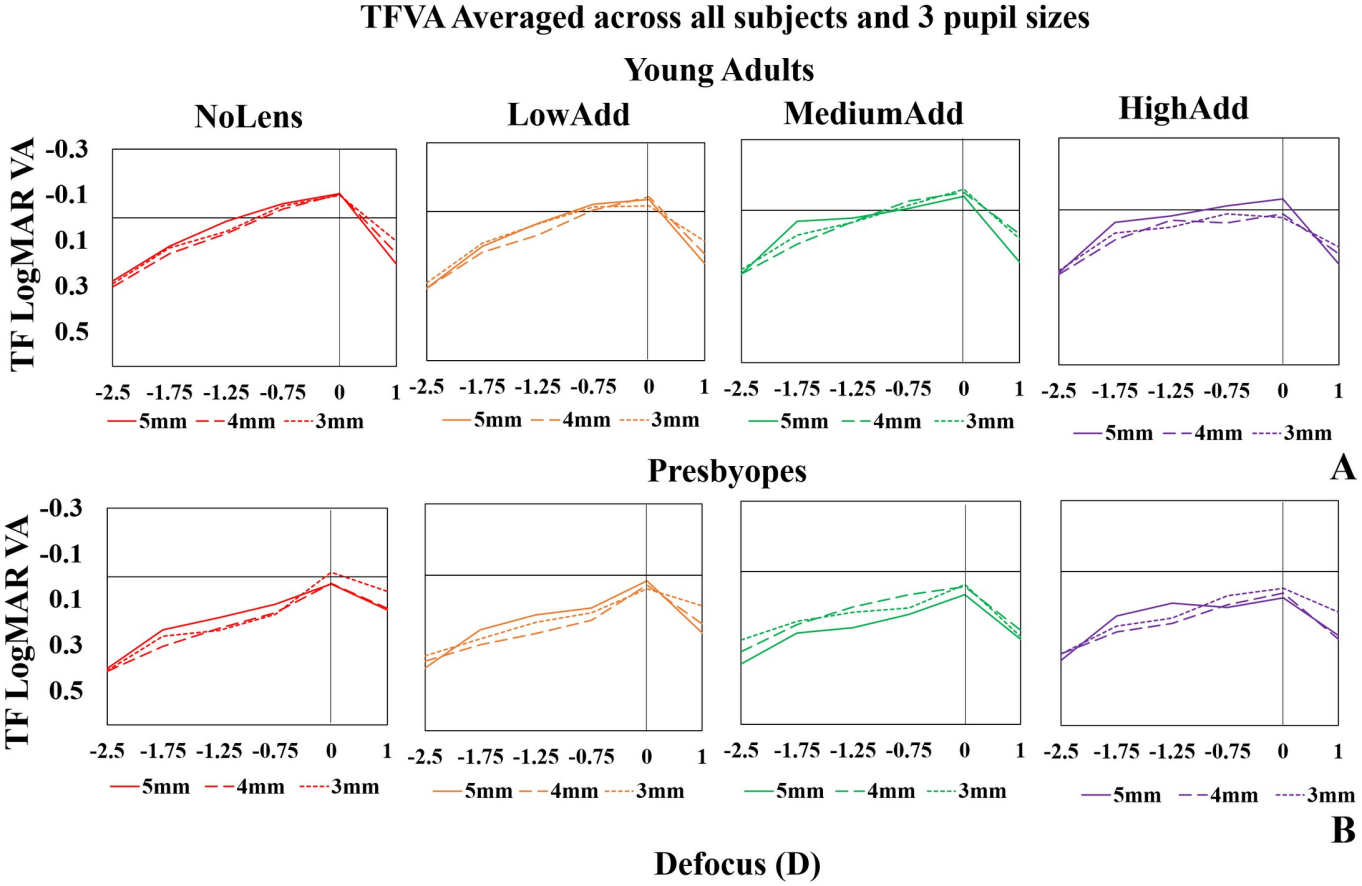
Average TF VA for 3 pupil diameters (3, 4 and 5-mm) under paralyzed accommodation. Lens condition identified by color—Red: NoLens; Orange: LowAdd; Green: MediumAdd; Purple: HighAdd. (A) young adults; (B) presbyopes.

Fig 6 shows TFVA measured under paralyzed accommodation and averaged across all subjects in young adults (A—upper panels) and presbyopes (B—lower panels) for different lenses (LowAdd: Orange; MediumAdd: Green; HighAdd: Purple and NoLens: Red) for three different pupil diameters (5, 4 and 3 mm). In young adults, VA at far was on average within 93% LogMAR for the three pupil diameters with the NoLens, LowAdd and MediumAdd lens conditions. VA at far improved with increasing pupil diameter for the HighAdd lens (0.08 logMAR shift from 3-mm to 5-mm pupil). Conversely, for presbyopes, VA at far was highest with the smallest pupil diameter in the NoLens condition (0.05 logMAR shift), Medium and HighAdd lenses (0.042 and 0.041 logMAR shifts).

Partial correlation coefficients between the TFVA curves of the 5-mm pupil with the 3- and 4-mm pupil diameters ranged between 0.464–0.797 and 0.548–0.806 respectively, and were statistically significant in all cases (p<0.001).

On average, across all lenses in young adults, DOF increased from 1.5D (3mm) to 2D (5 mm) (Partial correlation, p<0.001), and in presbyopes DOF increased from 1D (3mm) to 1.5D (5mm) (Partial correlation, p<0.001).

In young adults, the TFVA curves tend to be broader for all lenses with the largest pupil (by 42.14% on average). In presbyopes, however, the best performance with the MediumAdd lenses at all distances was found with the 4-mm pupil diameter, whereas for the HighAdd lenses, performance at best focus was better with the 3-mm lens, and the broader curve was found with the 5-mm pupils. Standard deviation across subjects ranged from 0.01 to 0.11.

### Visual acuity at far, intermediate and near

**Fig 7:**
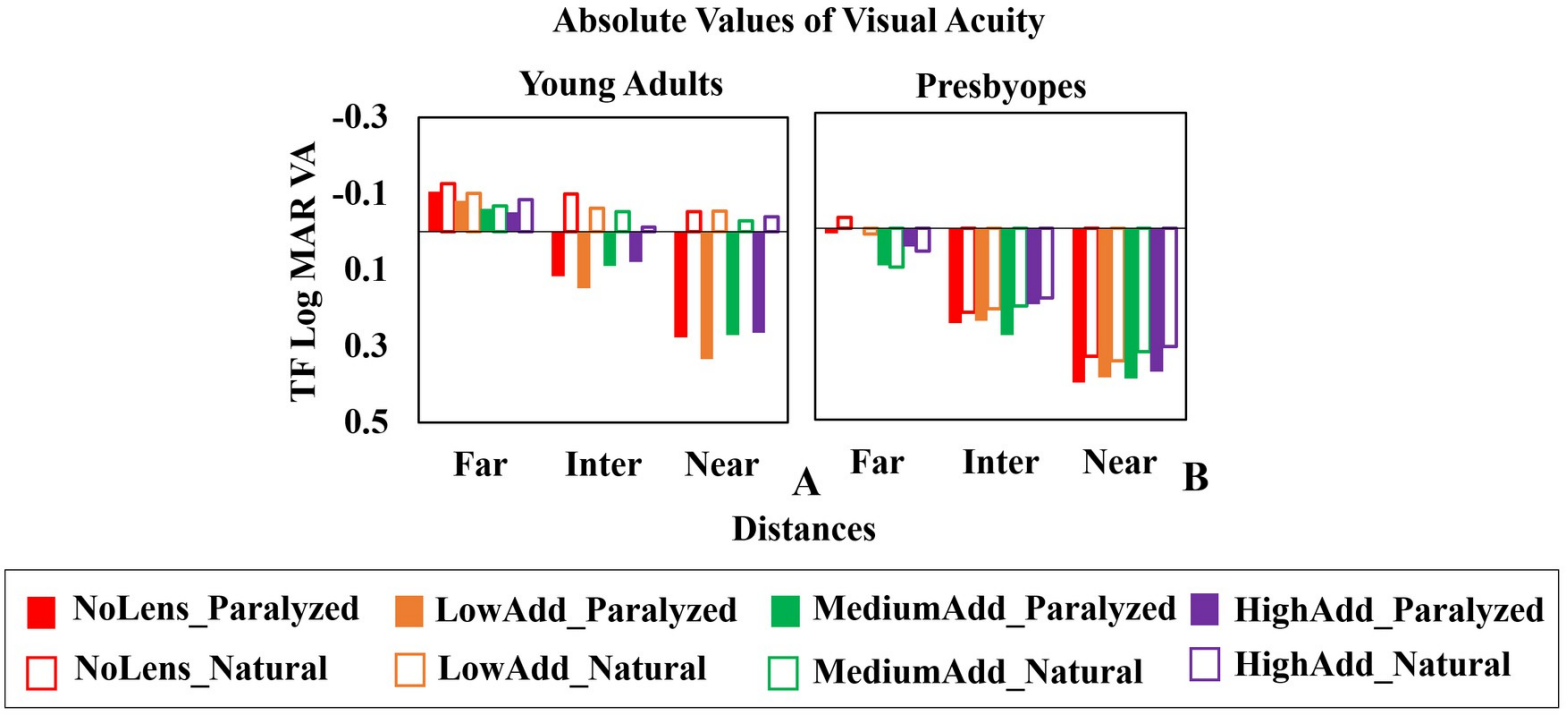
Average logMAR VA (across subjects) for far, intermediate, and near distances for both paralyzed accommodation and natural accommodation. (A) young adults. (B). presbyopes.–Red: NoLens; Orange: LowAdd; Green: MediumAdd; Purple: HighAdd; Solid bars: paralyzed accommodation; Open bars: natural accommodation.

We evaluated the average effect of the multifocal lenses on VA at far (0D), intermediate (1.5D) and near (2.5D) distances, obtained from the TF curve. Fig 7 shows average VA in young adults (A) and presbyopes (B) at those distances, for all lenses (LowAdd: Orange; MediumAdd: Green; HighAdd: Purple and NoLens: Red), both for paralyzed (solid bars) and natural (open bars) accommodation conditions.

For natural accommodation, VA was statistically significantly better in young adults than in presbyopes in all conditions at far (Mann-Whitney U test, NoLens p = 0.0008; LowAdd p = 0.008; MediumAdd p<0.001; HighAdd p = 0.003), intermediate and near (Mann-Whitney U test, NoLens/LowAdd/MediumAdd/HighAdd p<0.001). For paralyzed accommodation, VA was statistically significantly better in young adults than in presbyopes for far (Mann-Whitney U test, NoLens p = 0.013; MediumAdd p = 0.003 and HighAdd p = 0.019), intermediate (Mann-Whitney U test NoLens p = 0.019; MediumAdd/HighAdd p<0.001), and near (Mann-Whitney U test NoLens p = 0.028).

Young adults showed statistically significantly different VA values between paralyzed and natural accommodation for all conditions at intermediate (Wilcoxon signed rank test, p<0.013) and near (p<0.007), but not presbyopes. On average across conditions (lenses and accommodation status), VA at intermediate and near were worse than at far in all cases, by -0.11 and -0.18 logMAR for intermediate and -0.20 and -0.32 logMAR at near, in young adults and presbyopic patients respectively. In young adults under natural accommodation, VA was better than 0 logMAR in all conditions.

### Visual benefit at near and visual degradation at far

**Fig 8:**
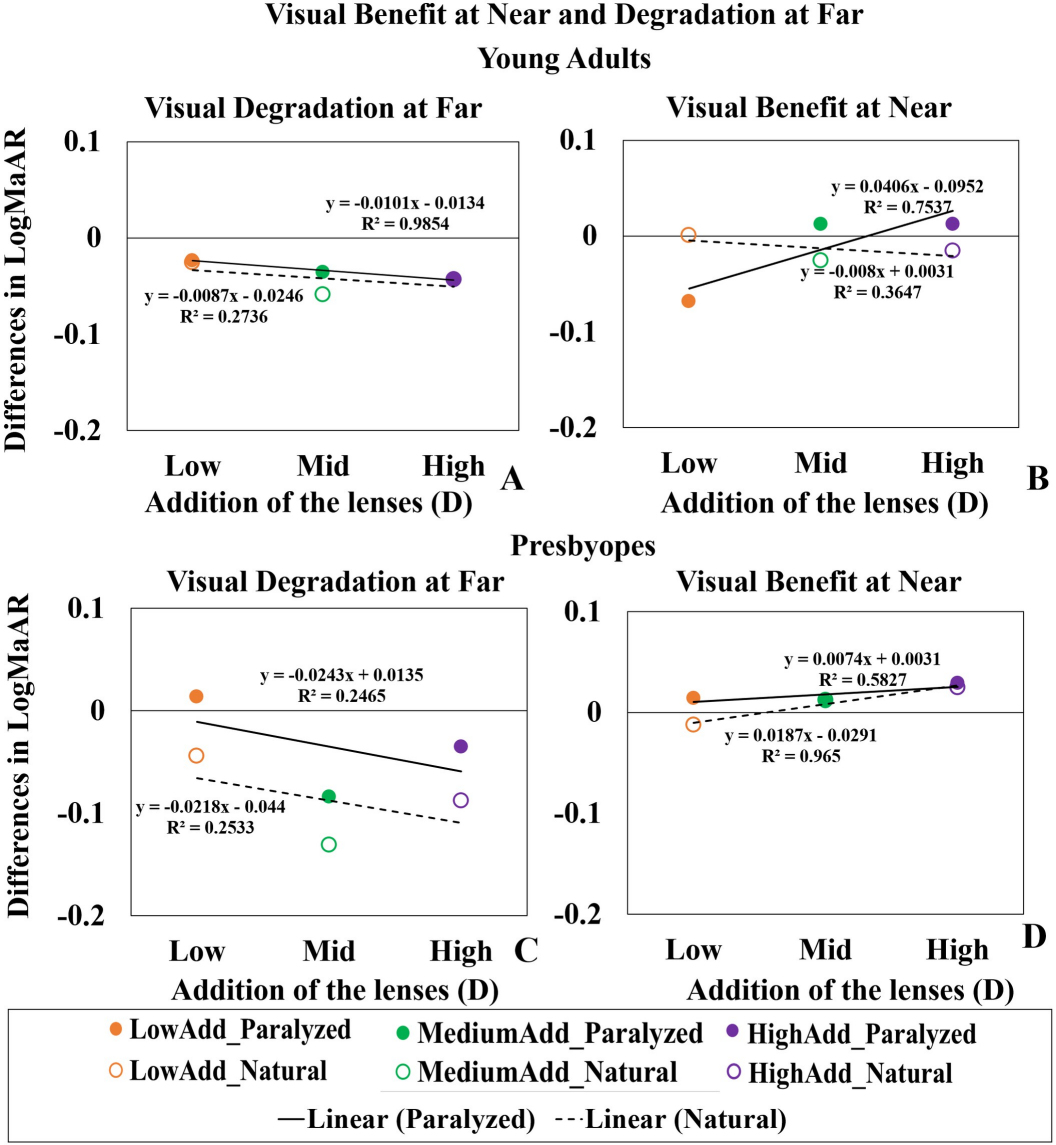
Visual benefit and visual degradation at far. (A), (C) Visual Degradation at far (left panels) and (B), (C)Visual Benefit at near (right panels) in young adults (upper panels) and presbyopes (lower panels), estimated as differences of logMAR VA with the MCLs and NoLens, averaged across subjects. Lens condition is identified by color—Orange: LowAdd; Green: MediumAdd; Purple: HighAdd. Accommodation condition is identified by fill pattern (solid symbols: paralyzed accommodation; open symbols: natural accommodation). Regression lines are shown as black solid lines for the paralyzed condition and dashed lines for the natural accommodation condition.

Fig 8 shows the visual degradation at far (Left panels, A and C) and the visual benefit at near (Right Panels, B and D) for young adults (upper panels) and presbyopes (lower panels) produced by all lenses (LowAdd: Orange; MediumAdd: Green; HighAdd: Purple) relative to the NoLens, as a function of the lens near add. Positive values in the y-axis represent a gain and negative values represent a loss in visual performance produced by the lens. Lines represent linear regression fitting of the data, as a function of near add values (slopes are in logMAR/Diopter of near add; and r stands for correlation coefficients).

On average, the MCLs produced a small but consistent degradation of far vision with increasing add in both groups and conditions (s = -0.013 logMAR/D, r = 0.98 in young adults; s = -0.03 logMAR/D, r = 0.24 in presbyopes), and a consistent benefit at near in the young adults with paralyzed accommodation (s = 0.06 logMAR/D, r = 0.75), and in presbyopic subjects with both paralyzed (s = 0.013 logMAR/D, r = 0.58) and natural accommodation (s = 0.029 logMAR/D, r = 0.96).

### Visual imbalance

**Fig 9:**
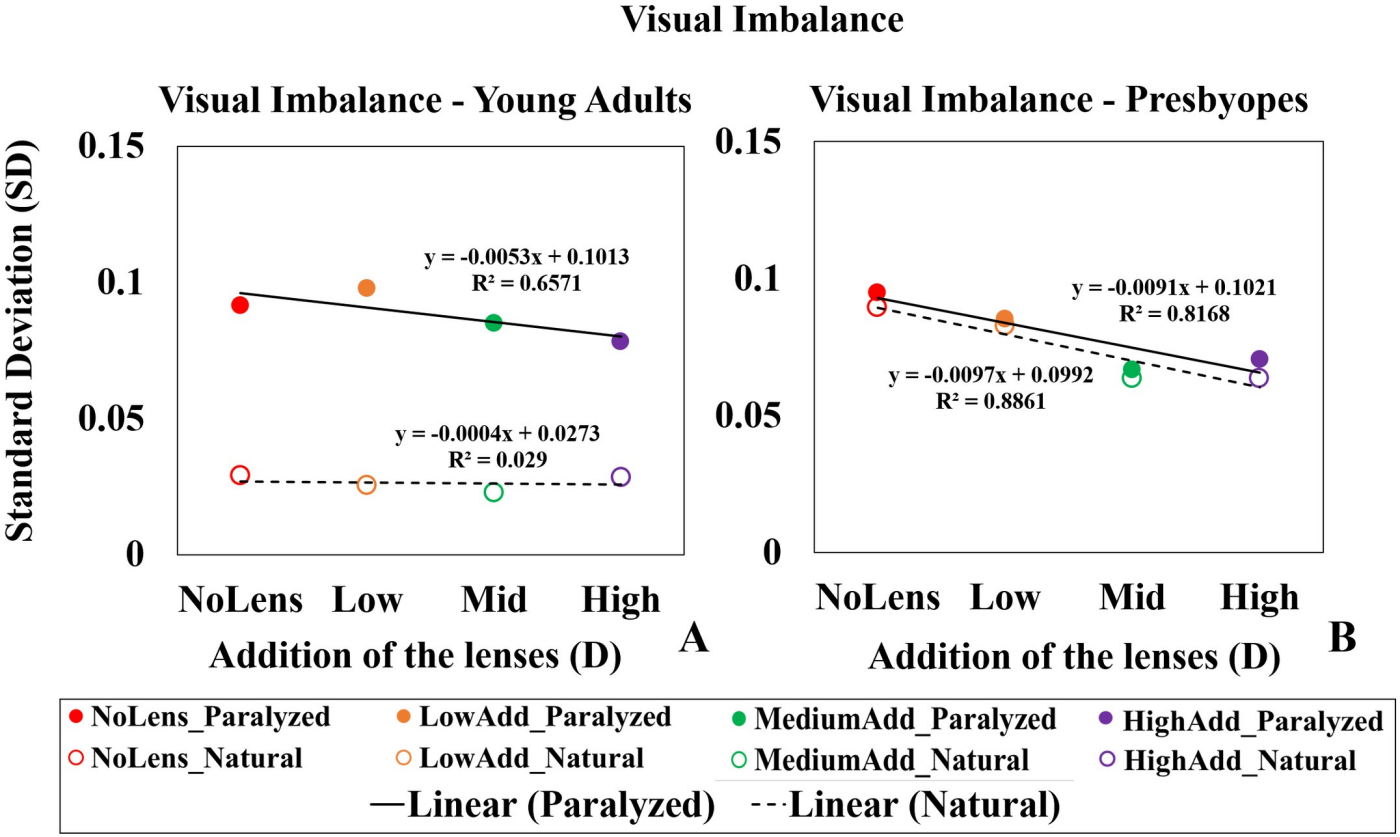
Visual imbalance across the VA curve. (A) young adults (B) presbyopes across paralyzed (solid symbols) and natural accommodation (open symbols) for different lenses, showing the averaged data (averaged across all subjects). (Lens condition identified by color—Red: NoLens; Orange: LowAdd; Green: MediumAdd; Purple: HighAdd). Regression lines shown as black solid lines for paralyzed condition and dashed lines for natural accommodation condition.

Fig 9 shows the visual imbalance metric, accounting for the variations in VA across the TF curve, in all conditions, as a function of near add, for young adults, (Fig 9A) and presbyopes (Fig 9B), showing averaged data for different lenses (NoLens: Red; LowAdd: Orange; MediumAdd: Green; HighAdd: Purple) across subjects and conditions (solid symbols: paralyzed accommodation; open symbols: natural accommodation).

Visual imbalance with natural accommodation was statistically significantly different between young adults and presbyopes in all conditions (Mann-Whitney U test, NoLens p = 0.001, LowAdd p<0.001, MediumAdd p<0.001 and HighAdd p = 0.003). Also, statistically significant differences in visual imbalance were found between paralyzed and natural accommodation in young adults in all conditions (Wilcoxon signed rank test, NoLens p = 0.005, LowAdd p = 0.007, MediumAdd p = 0.005 and HighAdd p = 0.009) but not in presbyopes.

On average, visual imbalance was reduced with increasing add in young adults with paralyzed accommodation (s = -0.06 D^-1^; r = 0.65) and in presbyopes with both paralyzed (s = -0.01 D^-1^; r = 0.81) and natural accommodation (s = -0.01 D^-1^; r = 0.88). The lowest imbalance occurred in young adults under natural accommodation, although even in this case, visual imbalance was reduced by 13.33% with MCLs with respect to the NoLens condition (averaged across the three near additions).

### Overall visual performance with MCLs

This analysis is generated by combining the visual degradation/benefit and visual imbalance metrics (normalized) as described in the previous sections. Higher the value higher the overall visual performance of the lens according to the defined metric. Although the metric can also reach negative values, it is noteworthy that overall visual quality was positive in all cases. The best overall performance was found in young adults under paralyzed accommodation (LowAdd:0.36;MediumAdd:0.37;HighAdd:0.35). In presbyopes (both under natural and paralyzed accommodation) the highest performance was found with LowAdd lenses (0.22). The overall visual performance metric with MCLs with paralyzed accommodation exceeded the overall performace with natural accommodation (by 76.2% in young adults and by 17.7% in presbyopes).

## Discussion

MCLs are well-known solutions for presbyopia. With MCLs becoming a popular strategy to stop myopia progression, evaluating visual quality with these lenses, and identifying various parameters affecting visual performance, becomes important. Although the current state-of-the art favors center-distance designs to treat myopia and specific lenses appear to target this market, some presbyopia MCL designs may be applicable in myopes. Thus, understanding fundamental differences in the performance of MCLs in both young adult and presbyopic populations is of interest.

Our study examined a center-near multifocal lens design and evaluated the effect of near add (low, medium and high) on visual acuity, as tested in two age groups (young adults and presbyopes) with natural and paralyzed accommodation. In order to reduce the number of variables in the study, all subjects used the same -2 D distance corrected contact lens (independent of their refraction), and the residual defocus was corrected with a Badal system. A previous study with the same MCLs calculated the TF optical performance of the multifocal lenses (based on their theoretical power profile), which was used as the basis for simulations of the lenses in a Simultaneous Vision Simulator [36, 45, 46] and a Spatial Light Modulator in an Adaptative Optics system [39]. TFVA was measured with the MCLs on the eye and the equivalent SimVis simulated lenses in presbyopic subjects. As in a prior study, in the current study we found that MCLs produced a decrease in far visual acuity as compared with a monofocal condition (NoLens), as expected in simultaneous vision corrections, where the energy is split into two foci and a blurred image is always superimposed onto a sharp component. Overall, we found a relatively large intersubject variability with paralyzed accommodation (Fig 5), and intra-subject differences, which varied with the lens design, paralyzed or natural accommodation (only in young adults), and to a lesser extent, pupil diameter (Fig 7). As a result of the age dependence of visual acuity, the maximally attained visual acuity (generally far vision with NoLens) in presbyopes was lower than that reached by the young adults.

An important parameter in multifocal lenses is the near add magnitude. We have tested all subjects with MCLs of three additions (Low: +1.25D; Medium: +1.75D; High: +2.50D). In clinical practice with presbyopes, the selection of addition is associated with the remaining accommodation amplitude, and therefore lower additions are prescribed to early presbyopes and higher additions to older presbyopes. The range of addition powers deemed satisfactory for prescribing MCLs to progressing myopes is large [3, 31, 32], with some lenses claiming inductions of up to +6 D near add [37]. Testing identical lenses (including the same base power) in all patients has allowed us to establish direct comparisons between subjects and between groups. On one hand, we found that the MCLs meet the purpose of gaining some near vision (compared to the NoLens case) in presbyopes or in simulated presbyopia (paralyzing accommodation) in young subjects. However, the MCLs did not improve near vision in the young adult group under natural accommodation, but maintained near vision from dropping significantly in comparison with the far VA, keeping vision fairly constant from far to near. Likewise, MCLs slightly degraded visual quality at far (up to 0.8 logMAR). However, in young adults, this drop did not degrade VA below 0 logMAR, therefore still providing good vision at far. The degradation was slightly larger for presbyopes, which along with a poorer baseline VA at far, resulted in VA poorer than 0 logMAR with all MCLs.

From previous studies, where we used simultaneous vision simulators to investigate the effect of near add in pure simultaneous vision visual acuity [5, 45] in patients with paralyzed accommodation, we found that far vision was not equally degraded with all near add magnitudes. Additions around +2.50D produced larger degradation than lower and higher additions, an effect that could be explained solely on optical grounds. We did not have the opportunity in the current study to test lenses with additions larger than +2.50 D. In any case, on average, the increase in visual degradation at far with near add found in the current study, both in young adults and presbyopes, is consistent with that previous finding i.e., with HighAdd the degradation at far was higher than the Low and Medium additions.

Previous clinical studies in young adults wearing MCLs reported various degrees of visual compromise. Fedtke et al [3] found a decrease in visual acuity with bifocal lenses as compared with single vision lenses, although the decrease was less with MCLs with negative spherical aberration (center-near design). On the other hand, Shah et al [31] found relatively higher high contrast visual acuities with concentric lenses and with center-distance, exceeding the visual acuities reported by Anstice & Philips, Walline et al and Fedtke et al [47]. Fedke et al measured a high contrast visual acuity of 0.10 ± 0.12 logMAR with a center-near MCLs with HighAdd, while the center-near LowAdd produced the best visual acuity overall, up to -0.05 ± 0.11 logMAR. In our experiments, we also observed that performance with the LowAdd lenses was more similar to the NoLens condition than higher add MCLs. In any case, we did not find that the center-near MCLs degraded vision significantly with respect to single vision, and therefore not using MCLs on the basis of a potentially poorer performance than center-distance MCLs (as suggested in prior work) [17] does not appear justified. Also, while differences in pupil diameter between young and presbyopic subjects have often been argued as the potential cause for different performance of center-near add MCLs in both groups, our findings show relatively small differences in TF performance with pupil diameter, suggesting that pupil diameter plays a secondary role in their performance.

The relative TF Visual performance with paralyzed accommodation is similar in young adults and presbyopes (also in presbyopes with natural accommodation), with consistent performances with increasing near add, indicating that the lens designs prevail over other factors. With natural accommodation, in the same subjects, the curves widen, revealing that accommodation, and not only the lens design, plays a role in the MCL performance in young adults. This could be achieved by fine-tuning the near peak of accommodation to bring the near target in focus with a lower accommodative demand. However, it is most likely that young adults accommodate the full range using the far peak, with the defocused image of the near focus always out of focus (by different amounts). In this case, the combination of a center-near and the natural change of spherical aberration with accommodation could contribute to decreasing accommodative lag and improving performance at near. In our study most of the subjects with Medium and HighAdd lenses (Fig 4) appeared to use the far peak to accommodate for the entire range of tested near vision. Only two subjects (S#3, S#10) appeared to fail to accommodate the full range.

Our results show that MCLs produce a more uniform quality of vision across distances. We can speculate that a higher visual constancy is a preferred scenario than overall better (but also less uniform) vision, as achieved in young adults with natural vision, and this may explain the high degree of acceptance of MCLs when prescribed to myopes. Although the selection of multifocal lenses in myopes should be based on their success to control myopia, excellent vision at near and far cannot be compromised. The overall visual quality metric, which takes into account other parameters such as visual constancy, can be used to aid in the selection of the optimal lens design on the basis of performance. In fact, the use of new Simultaneous Vision Simulators would allow non-invasive testing of a range of designs without physically putting them on the eye [36, 45, 48]. The relatively large intersubject variability makes it advisable to provide patients (both young and presbyopic) the experience of multifocality before the fitting.

In summary, the present study reveals that center-near lenses do not degrade high contrast visual acuity significantly while maintaining far vision in young adults and produce some visual benefit at near in presbyopes. Factors such as visual degradation at far, visual benefit at near and visual constancy (uniformity of vision across distances) should be considered in the management of myopia and presbyopia with MCLs and to gain insights on the mechanism of operation of these lenses. Future studies on visual quality with MCLs may include contrast sensitivity and the Multifocal Acceptance Score metric [44, 49], which uses natural scenes for far/near and day/night conditions in an attempt to assess perceptual quality with real-life images beyond high contrast VA.

## Conclusions

- VA was generally better in the young adult group than the presbyopic group for all conditions (NoLens and MCLs)
- In young adults, the shape of the TFVA curve was significantly different between paralyzed and natural accommodation conditions. In contrast, presbyopes did not show significant differences between paralyzed and natural accommodation, as expected due to lack of accommodation in the older group.
- The visual imbalance (variations of VA constancy from far to near) decreased with MCLs, in comparison with the NoLens. This result is consistent with an increase in the negative spherical aberration with the center-near design, which has a favorable effect in decreasing accommodative lag
- The MCLs produced a small but consistent degradation at far in all conditions, and a consistent benefit at near in young subjects with paralyzed accommodation and in presbyopic subjects with both paralyzed and natural accommodation. Visual degradation at far increases with more addition in the lenses.
- Under paralyzed accommodation, both in presbyopes and young adults, the depth of focus of the TF curve increased with increasing pupil diameter (3mm to 5mm).

## Supporting information

S1 File (DOCX)
