# Supplementary material for: Visual performance with multifocal lenses in young adults and presbyopes
Source: PLoS One. 2022 Mar 17;17(3):e0263659. doi: 10.1371/journal.pone.0263659 (PMC8929584; doi:10.1371/journal.pone.0263659)
Supplement: S1 File — (DOCX) [file pone.0263659.s001.docx]

| Figure | Statistical method | P value | samples |
| --- | --- | --- | --- |
| **Fig 2**  1) Across conditions  2) Far vs Near in presbyopes  3) Far vs Near in young adults  4)Between two subject groups | Kruskal-Wallis test  Wilcoxan signed Rank test  Wilcoxan signed Rank test  Mann-Whitney U test | *p<0.05  *p<0.05  *p<0.05    *p<0.05  *p<0.05 | 10  5  5    10  15 |
| **Fig 4**  1)Between paralyzed and natural state | Wilcoxan signed Rank test | *p<0.05 | 10 |
| **Fig 6**  1)Shape of the TFVA curves between natural and paralyzed state | Shape similarity metric: cross correlation | *p<0.05  p>0.05 | 10  5 |
| **Fig 7**  1)Pupil diameter correlation for 5mm,4mm and 3mm | Partial correlation coefficients | *p<0.001  *p<0.001 | 10  5 |
| **Fig 8**  1)Between two subject groups  2)Across distances (far, intermediate, near) between subject groups  3) Between paralyzed and natural state at different distances | Mann-whitney U test  Mann-whitney U test  Wilcoxan signed Rank test | *p<0.001  *p<0.05  *p<0.05 | 15  15  10 |
| **Fig 10**  1) Between two subject groups  2) Between paralyzed and natural state | Mann-Whitney U test  Wilcoxan signed Rank test | *p<0.05    *p<0.05 | 15    10 |
